# Consensus statement: a framework for safe and effective intubation by paramedics

**DOI:** 10.29045/14784726.2018.06.3.1.23

**Published:** 2018-06-01

**Authors:** Paul Gowens, Paul Aitken-Fell, Will Broughton, Liz Harris, Julia Williams, Paul Younger, David Bywater, Colin Crookston, Lisa Curatolo, Tim Edwards, Els Freshwater, Matt House, Andy Jones, Mark Millins, Richard Pilbery, Simon Standen, Christian Wiggin

**Affiliations:** College of Paramedics; College of Paramedics; College of Paramedics; College of Paramedics; College of Paramedics; College of Paramedics; Scottish Ambulance Service; Scottish Ambulance Service; Scottish Ambulance Service; Emergency Medical Retrieval Service Scotland; London Ambulance Service; Hampshire and Isle of Wight Air Ambulance; University Hospital Southampton; North West Ambulance Service; Welsh Ambulance Service; Yorkshire Ambulance Service; Yorkshire Ambulance Service; Yorkshire Ambulance Service; South Western Ambulance Service

**Keywords:** paramedics, tracheal intubation

## Abstract

This consensus statement provides profession-specific guidance in relation to tracheal intubation by paramedics – a procedure that the College of Paramedics supports.

Tracheal intubation by paramedics has been the subject of professional and legal debate as well as crown investigation. It is therefore timely that the College of Paramedics, through this consensus group, reviews the available evidence and expert opinion in order to prevent patient harm and promote patient safety, clinical effectiveness and professional standards.

It is not the purpose of this consensus statement to remove the skill of tracheal intubation from paramedics. Neither is it intended to debate the efficacy of intubation or the effect on mortality or morbidity, as other formal research studies will answer those questions.

The consensus of this group is that paramedics can perform tracheal intubation safely and effectively. However, a safe, well-governed system of continual training, education and competency must be in place to serve both patients and the paramedics delivering their care.

## Background

Tracheal intubation (TI) has been considered a core skill for all paramedics since the 1980s and continues to be taught within the majority of pre-registration paramedic training programmes. With the introduction of supra-glottic airway devices (SADs), guidance provided by both the Joint Royal Colleges Ambulance Liaison Committee (JRCALC) and the Resuscitation Council (UK) has shifted emphasis to the primary use of such devices rather than intubation in cardiac arrest.

In the transition from vocational Institute of Health and Care Development (IHCD) national paramedic training to pre-registration programmes delivered within Higher Education Institutions (HEIs), the standards of both training and assessment of competence in TI now vary considerably; this has been compounded by reduced opportunities for supervised clinical practice within the operating theatre environment.

Since the introduction of SADs and the increased application of recognition of life extinct (ROLE) guidance, the number of intubations performed by paramedics has decreased. A self-reported survey of 1056 paramedics in the United Kingdom states a median of three intubation attempts per paramedic, per year (interquartile range 1–5, range 0–11) (Younger, Pilbery, & Lethbridge, 2016).

Paramedic intubation in the United Kingdom is generally undertaken without the administration of drugs. As a result of this, the majority of patients who are intubated will either be in respiratory or cardiac arrest. The optimal strategy for managing the airway in out-of-hospital cardiac arrest (OHCA) remains equivocal.

Paramedics work in a variety of clinical services and settings, including NHS Ambulance Services, hospital and acute care settings, primary and urgent care, the military, private ambulance providers and remote, off-shore and event medicine. The opportunities to practise TI may vary across each setting, as may opportunities for on-going training and assessment of competence. Where TI remains part of a paramedic’s scope of practice, employers must provide support to maintain competency.

## Definition of terminology

### Intubation attempt:

The insertion of a laryngoscope into the airway cavity to gain a view of the laryngeal inlet, with the intention of passing a tracheal tube through the inlet and into the trachea.

### Successful intubation:

The passage of a tracheal tube through the vocal cords and into the trachea with confirmation of tube placement by clinical assessment and objective monitoring (capnography).

### Failed laryngoscopy:

Failure to achieve an adequate view of the laryngeal inlet/vocal cords.

### Failed intubation:

Failure to pass an endotracheal tube into the trachea.

### Simulation:

Any technique that invokes or replicates substantial aspects of the real world, in a fully interactive manner (Gaba, 2004).

## Review of the available evidence

Out-of-hospital airway management has a profound effect on mortality and morbidity, and is a fundamental part of routine paramedic practice. Historically, TI has been the cornerstone of invasive airway management by para-medics (Woollard & Furber, 2010). However, recent research has questioned the value of this technique when performed by practitioners with comparatively less extensive training and relatively limited procedural exposure (Deakin et al., 2010).

A meta-analysis of pre-hospital airway management reported pooled procedural success rates for TI of 86.3% (95% CI 82.6%–89.4%) for non-physician providers globally, including paramedics (Hubble et al., 2010). Limited evidence exists in relation to UK paramedics, with retrospective reviews of TI conducted in an ambulance service and air ambulance system reporting procedural success rates of 83.8% (n = 368) and 97.3% (n = 36) respectively. Some investigators report improved survival where TI is performed by paramedics in cardiac arrest cases (McMullan et al., 2014), whereas others have observed increased mortality (Hanif, Kaji, & Niemann, 2010). Other research suggests that the aetiology of the cardiac arrest (Takei, Enami, Yachida, Ohta, & Inaba, 2010) or the sequencing of the procedure with other resuscitation tasks may be important factors in predicting the therapeutic value of endotracheal intubation (ETI) over other airway management approaches (Turgulov, Rac, Kierzek, & Morrison, 2011). Levels of practitioner education, experience and procedural exposure are also likely to influence outcomes (Dyson et al., 2016, 2017; Gold & Eisenberg, 2009).

A rapid evidence review was commissioned by the College in order to inform the consensus group (Pilbery, 2018). This evidence review specifically considered the question: ‘How do paramedics learn and maintain the skill of tracheal intubation?’. Learning, developing and maintaining competence is crucial to patient safety.

As a result of the evidence synthesis of this rapid evidence review, the consensus group has made the following observations:

1. In order to determine how paramedics learn and maintain the skill of intubation, a definition is required so it is clear what benchmark is to be used to state that a paramedic has ‘learnt’ the skill of intubation. This could be a range of measures, such as intubation success and complication rates, laryngoscopy technique and decision-making.
2. The precise number of intubations required to become proficient is not clear, but based on the evidence in the review, in order to achieve a first-pass intubation success rate of 90%, paramedic students require in excess of 25 intubations on patients, preferably in a range of environments (e.g. commencing in operating theatres or other in-hospital settings, and then out-of-hospital).
3. Education and training in TI should utilise a range of modalities, including didactic lectures, videos and practical skill stations and simulation. Non-technical skills must also be included within the syllabus. Students should not be trained on only a single manikin, but should have access to multiple types. Supervision by experienced faculty is required.
4. There is a paucity of evidence about how paramedics maintain their skill in intubation, given the lack of clinical opportunity in some settings. Retention can be supported by demonstrations and lectures by experienced faculty and opportunities for supported simulated practice, desirable on a yearly basis.

Further research is required to understand how paramedics maintain their skill in intubation, given the limited opportunities to use the skill in a clinical setting and lack of opportunities with employing organisations for retraining.

## Laryngoscopy in managing foreign body airway obstruction

All paramedics must be proficient in the use of a laryn-goscope and the technique of direct laryngoscopy with concomitant use of Magill forceps to facilitate the removal of a foreign body airway obstruction (FBAO). Laryngoscopy for airway clearance in cases of FBAO differs from laryngoscopy to facilitate passage of a tube into the trachea. Paramedic intubation Significant on-going clinical exposure is required to develop and maintain competence after completion of initial training in TI. The College recognises that there is a need for advanced airway management in paramedic practice and recommends that where paramedic intubation is performed, employers should provide a higher level of training, including simulated practice, supervised clinical practice and continued rigorous assessment of competence.

It is acknowledged that system configuration will necessarily vary according to population demographics and geographical considerations. For example, rural services may require different approaches to ensure that intubation-competent practitioners are universally available for the patient who requires advanced airway management.

## Standards of education and training

Laryngoscopy for the purposes of managing FBAO should remain a core skill and competency within all pre-registration paramedic training programmes. The skill of intubation should also continue to be taught, as it is important that all paramedics understand the procedure and have the ability to undertake an assisting role. The decision to undertake formative and summative assessments will be the responsibility of education providers and, where they are able to access appropriate clinical placements (e.g. operating theatres), this practice is encouraged and should continue.

Pre-registration training must include teaching of non-technical skills relating to the team approach to intubation and the use of essential equipment including bougies and capnography. Post-registration training of paramedics who undertake intubation must include comprehensive training in failed intubation drills and consider the use of a wider range of tools designed to improve laryngoscopy in difficult cases. Post-registration education and training must also include team skills and complex decision-making skills in relation to the clinical risks versus benefits of intubation.

Paramedics who practise TI maintain a shared responsibility with their employing organisations to ensure regular access to training, supervised practice and assessment of competence. A programme of on-going training and assessment must be evidenced to ensure that paramedics remain competent following initial training.

## Assessment of competence

There is significant variation within the evidence relating to the development of competence to undertake intubation. Traditional paramedic training used a benchmark standard of 25 supervised intubations in a controlled, theatre environment. However, since the transition to HEI-delivered paramedic training there is now no mandated national standard.

Assessment of competence needs to test the capability of paramedics in the psychomotor skills required to undertake safe and effective laryngoscopy and successful TI, and the essential non-technical skills pertaining to decision-making, risk assessment and leading the team involved. Assessment of competence should also include assessment of the paramedic’s ability to systematically troubleshoot difficulties in obtaining a laryngoscope view and in the use of failed airway drills.

While supervised practice in a controlled environment is essential in the development of competence, the use of simulation allows the development of competence not only in the technical, but also the non-technical skills related to undertaking intubation and associated decision-making, particularly in the pre-hospital setting. The use of simulated practice is encouraged at all levels. It is the consensus of this group that paramedics should expect to undertake 60 supervised intubations before being deemed competent, of which a minimum of 25 must be undertaken in a controlled setting (i.e. hospital anaesthetic room). Simulation in a context-specific environment may be utilised to achieve the total number of supervised intubations. Assessment of competence needs to include demonstration of safe practice in managing complications and in the use of failed airway drills.

Following an initial assessment of competence, all paramedics who undertake intubation must maintain an airway management log, which must include all intubation attempts, including training and simulation in addition to operational practice. It is the consensus of this group that a minimum of two intubations per month should be logged, for each patient age group for which intubation is permitted (infant, child and adult); these can be in clinical practice or in a simulation setting. Airway logs should allow for the recording of information about the circumstances of the case and should record aspects of decision-making and reflection on both the technical and non-technical skills used.

Annual refresher training and assessment of competence must take place and should include simulation that facilitates assessment of both technical and non-technical skills. Formal review of airway logs should also be undertaken as part of the annual review process.

## Standards of equipment

It would not be appropriate for this consensus statement to mandate the type and volume of equipment that organisations should provide to the paramedic workforce to allow intubation to be undertaken, beyond stating that the provision of a bougie and use of capnography are mandatory minimum standards that must be applied routinely by all paramedics performing TI.

Organisations should also consider the provision of and training in additional methods designed to improve the safety of intubation, and should include equipment and training necessary to facilitate rescue airway drills.

## Clinical governance

Organisations must ensure that robust systems of clinical governance are in place, including the following:

- A named clinician with lead responsibility for the practice of intubation by paramedics.
- A clearly defined clinical governance policy and framework that outlines the systems in place to monitor and assure quality and safety.
- Regular audits of capnography use, including a feedback mechanism to staff.
- Documents that guide practice (e.g. policies, standard operating procedures, guidelines and checklists).
- Defined standards of practice, supported by clinical audit, which must include the mechanisms by which audit findings will be addressed.
- Defined processes for education, training and assessment of competence and the processes in place for maintenance and reassessment of competence.
- Education and training of staff undertaking support roles, such as the airway assistant role.
- Standardised equipment, which must include the provision of bougies, waveform capnography and any other items deemed necessary to maintain safe systems of work.
- Defined processes for adverse event and incident reporting and how the learning from such events is translated into operational practice.

## Summary and conclusion

While TI has been successfully undertaken by paramedics for many years, it remains the subject of contention and debate. It is the consensus of this group that the development of SADs has reduced exposure to the use of this skill in practice, raising some concern over the ability for an individual paramedic to maintain competency in this skill.

There is variety in the education, training and assessment of competence and the provision of equipment to facilitate TI, with no nationally agreed standard. There are also no formal standards regarding the continuing assessment of competence. The College will work to put the necessary guidance in place.

Laryngoscopy and the concomitant use of Magill forceps is a core skill for all paramedics in the management of FBAO. The continued practice of TI by para-medics for the benefit of the patients requires organisations to work with its paramedics to ensure this clinical skill is performed safely.

Organisations must ensure that they have appropriate clinical governance processes in place to support this activity, with a named individual identified and accountable for clinical practice, and that robust processes are in place for the audit and review of practice and the maintenance of staff competence.

It is the consensus of this group that this statement sets out the high standards for safe and effective TI by paramedics.

## Conflict of interest

None declared.

## Funding

None.
